# Metabolic syndrome in Thai adolescents and associated factors: the Thai National Health Examination Survey V (NHES V)

**DOI:** 10.1186/s12889-021-10728-6

**Published:** 2021-04-07

**Authors:** Sirinapa Siwarom, Wichai Aekplakorn, Kwanchai Pirojsakul, Witchuri Paksi, Pattapong Kessomboon, Nareemarn Neelapaichit, Suwat Chariyalertsak, Sawitri Assanangkornchai, Surasak Taneepanichskul

**Affiliations:** 1 Department of Pediatrics, Faculty of Medicine Ramathibodi Hospital, Mahidol University, Bangkok, Thailand; 2 Department of Community Medicine, Faculty of Medicine Ramathibodi Hospital, Mahidol University, 270 Rama VI Road, Thung Phayathai, Ratchathewi, Bangkok, 10400 Thailand; 3 Department of Community Medicine, Faculty of Medicine, Khon Kaen University, Khon Kaen, Thailand; 4 Ramathibodi School of Nursing, Faculty of Medicine Ramathibodi Hospital, Mahidol University, Bangkok, Thailand; 5 Faculty of Public Health, Chiang Mai University, Chiang Mai, Thailand; 6 Epidemiology Unit, Faculty of Medicine, Prince of Songkla University, Songkhla, Thailand; 7 College of Public Health Sciences, Chulalongkorn University, Bangkok, Thailand

**Keywords:** Metabolic syndrome, Adolescents, Prevalence, Risk factors

## Abstract

**Background:**

Presence of metabolic syndrome (MetS) in early life may influence cardiovascular outcome later in adulthood. There is limited data regarding MetS among Thai adolescents. This study aimed to estimate the prevalence of MetS and related factors in Thai adolescents.

**Methods:**

Data on MetS components of 1934 Thai adolescents aged 10–16 years were obtained from the 5th National Health Examination Survey. Age at first screen time exposure, duration of screen time, frequency of food intake and physical activities were collected from interviews. MetS was defined according to 3 definitions: International Diabetes Federation (IDF), Cook’s, and de Ferranti’s.

**Results:**

The prevalence of MetS was 4.1% by IDF, 8.0% by Cook’s, and 16.8% by de Ferranti’s definition. The overall prevalence was higher in male (19.0%) than female adolescents (15.3%). The most common MetS components composition among Thai adolescents was high waist circumference with high serum triglyceride and low HDL-cholesterol (40.0% for IDF, 22.6% for Cook’s and 43.5% for de Ferranti’s definition). Exposure to screen media during the first 2 years of life had a 1.3- fold increased odds of MetS by 1 out of 3 definitions (OR 1.30, 95% CI. 1.01–1.68). Duration of physical activity associated with decreased odds of MetS by Cook’s definition (OR 0.96, 95% CI. 0.92–0.99).

**Conclusions:**

The prevalence of MetS among Thai adolescents was higher than previously reported by other studies. Screen media exposure during the first 2 years of life should be discouraged and measures to promote physical activity among children and adolescents should be strengthen.

**Supplementary Information:**

The online version contains supplementary material available at 10.1186/s12889-021-10728-6.

## Background

Cardiovascular disease is a leading cause of mortality and morbidity worldwide and it contributed to over 120,000 deaths or 23% of all deaths in Thailand in 2016 [1]. Metabolic syndrome, a cluster of cardiometabolic risks, has been shown to be associated with coronary heart disease in adults [2]. Many studies suggested that childhood metabolic risk factors are associated with adult cardiovascular disease. Atherosclerosis begins in childhood and progresses to more advanced stages during adulthood [3, 4]. Metabolically unhealthy children and adolescents have tendency to become adults with higher cardiovascular risk later in life [5–9]. Early identification with appropriate treatment and health supervision in these adolescents with multiple risk factors are important in lowering their future cardiovascular risk.

There is currently no universal definition of metabolic syndrome in children and adolescents. The available definitions are based on five common cardiometabolic risk factors including abdominal obesity, high level of serum triglyceride, high fasting blood glucose, high blood pressure and low plasma HDL cholesterol (HDL-C). The commonly used pediatric definitions of metabolic syndrome include those adapted from the Third report of National Cholesterol Education Program expert panel on detection, evaluation, and treatment of high blood cholesterol in adults (NCEP-ATP III) criteria such as Cook’s [10] and de Ferranti’s [11] definitions. Both of these two definitions require 3 out of 5 previously mentioned components, but with different cut-off levels. Another widely used definition is from the International Diabetes Federation (IDF), which requires abdominal obesity as a mandatory criterion plus 2 out of the other 4 components to define metabolic syndrome, again with some differences in the cut-off levels from other definitions [12].

There are few studies that have reported the prevalence of metabolic syndrome in children and adolescents in Thailand. A cross-sectional study in Grade 1–9 schoolchildren from a single district in 2009 revealed the metabolic syndrome prevalence by de Ferranti’s definition of only 4% in the study population of 348 children [13]. However, serum HDL-C was not evaluated in the study. Another study in a different province among 393 adolescents aged 13–16 years during 2013–2014 showed the prevalence of metabolic syndrome of 3.5, 5.8, and 11.2% according to IDF, Cook’s, and de Ferranti’s definition respectively [14]. Both of the studies were limited to a district and a provincial level. Currently, there has been no study evaluating the prevalence of metabolic syndrome among Thai adolescents on the national level. The present study used the data from the National Health Examination Survey in 2014 to estimate the prevalence of metabolic syndrome and determine life style factors that related to the condition in Thai adolescents.

## Methods

The 5th Thai National Health Examination Survey (NHES V) was a large-scale survey carried out all over the country in 2014. The survey included 32,400 participants of all ages starting from 1 year old from five regions including Bangkok, Central region, Northern region, North-eastern region, and Southern region. For this study, adolescents aged 10–16 years from Thai NHES V survey were included. Metabolic syndrome components including waist circumference, systolic and diastolic blood pressure, serum triglyceride, serum HDL-C, and fasting blood glucose were analysed to classify metabolic syndrome according to 3 different definitions: IDF, Cook’s, and de Ferranti’s.

All metabolic syndrome components were measured using standardized techniques. Waist circumferences were measured twice in each participant at midpoint between the lowest rib and iliac crest using non-elastic tape to the closest 0.1 cm, the average between the two measurements were used for analysis. Blood pressures were measured in sitting position after a 5-min rest. Each participant had 3 measurements of blood pressure recorded at 1-min intervals, the first reading was discarded and the average between the second and third readings were used for analysis. Blood collection for fasting blood glucose, HDL-C and triglyceride were performed after a 12-h fast.

The demographic data including age, sex, caretaker’s education level and income were collected from interviews. Body weight was measured using digital weighing scale with 0.1 kg accuracy. Height was measured using standard metal tape with 0.1 cm accuracy. Weight for age z-score, height for age z-score, and weight for height z-score were calculated according to national Thai growth reference. The World Health Organization’s growth standard was used to determine body mass index (BMI) z-score for age and sex. Obesity was defined by BMI z-score of > 2 and overweight by BMI z-score of > 1 and ≤ 2. Waist circumference percentile curves for Malaysian children and adolescents [15] were used as reference for calculation of waist circumference percentiles for age and sex. Data on intake of foods high in sugar and saturated fat including sweetened milk, soft drinks and other sugar sweetened beverages, and dessert with coconut milk were derived from food frequency questionnaire [16]. Daily amount (serving) of fruits and vegetables consumption was estimated based on separate questions. Age at onset of screen media exposure, screen time duration including television watching, computer, smart phone, tablet use, and time involved in physical activities were also obtained from interviews. The participants and their parent or caretaker took part in the interview process together. The age at onset of screen media exposure was categorized into two groups using the cut-off age of 2 years old to represent early life screen exposure. The cut-off age was chosen according to the screen time recommendation by the American Academy of Pediatrics, which suggested avoiding screen media for children up to age 2 years.

### Statistical analysis

Descriptive statistics including prevalence, proportion, mean, and standard deviation were used. Prevalence of metabolic syndrome by each definition was calculated. Cohen Kappa was used to measure the degree of agreement between each pair of metabolic syndrome definitions. The characteristics of participants with and without metabolic syndrome were compared using chi-square test for categorical variables and t-test or Kruskal Wallis for ratio scale where appropriate. Percentages of each and clustering of metabolic syndrome components among male and female participants were compared using chi-square test. Multivariable logistic regression analysis was performed to determine association between risk factors and metabolic syndrome. In the logistic regression model, independent variables included age, sex, food intake of each item (> 3 vs ≤3 times/week), total screen time (hour/week), screen media exposure during the first 2 years of life (yes/no), physical activity (hour/week), fruit and vegetable intake (portion per day). Odds ratio and 95% confidence interval (CI) were reported. All statistical analysis was done using IBM SPSS Statistics software version 22.

## Results

There were 2831 children aged 10–16 years in the NHES V study. A total number of 1934 participants (68.3%) were included in this analysis and 897 children were excluded due to incomplete glucose and lipid data. The number of participants who had metabolic syndrome according to at least one of the three definitions was 332 (17%). The participants who had metabolic syndrome by at least one definition were slightly younger than those without metabolic syndrome. There were significantly higher proportion of male sex, obesity, and overweight among participants with metabolic syndrome. Weight-for-age z-score, height-for-age z-score, weight-for-height z-score, BMI z-score, waist-to-height ratio, fasting blood glucose, serum triglyceride, and serum LDL-C levels were significantly higher in participants with metabolic syndrome compared to those without metabolic syndrome. Serum HDL-C levels were significantly lower in participants with metabolic syndrome compared to those without. There were no statistically significant differences in the duration of physical activities, screen time, onset of screen media exposure, daily amount of fruits and vegetables intake, frequent intake of sweetened milk, soft drinks or other sweetened beverages, and dessert with coconut milk between the two groups. The characteristics of the participants are shown in Table 1.

**Table 1:** Baseline characteristics

Parameter	Total (*N* = 1934)				MetS^a^ (*N* = 332)				No MetS (*N* = 1602)				***p***-value
	N	%	Mean	S.D.	N	%	Mean	S.D.	N	%	Mean	S.D.	
**Age** (year)			13.40	1.94			13.05	1.88			13.47	1.94	< 0.001
**Sex** (N (%))													
Male	973	50.3			185	55.7			788	49.2			0.035
Female	961	49.7			147	44.3			814	50.8			
**Caretaker’s education**													
Primary school and below	873	45.1			149	44.9			724	45.2			0.961
Secondary school and diploma	636	32.9			104	31.3			532	33.2			
Bachelor degree and above	189	9.8			37	11.1			152	9.5			
Other/data not available	236	12.2			42	12.7			194	12.1			
**Family income**													
Lower income than expense	413	21.4			72	21.7			339	21.2			0.551
Having debt	967	50.0			166	50.0			801	50.0			0.997
No saving	428	22.1			89	26.8			341	21.2			0.072
**Anthropometric classification**													
Obesity (BMIZ^b^ > 2)	296	15.3			183	55.1			113	7.1			< 0.001
Overweight (2 ≥ BMIZ > 1)	164	8.5			58	17.5			106	6.6			< 0.001
Thinness (BMIZ < −2)	4	0.2			0	0			4	0.2			1.000
Stunting (HAZ^c^ < −2)	39	2.0			3	0.9			36	2.2			0.134
**Food intake**													
Frequent (> 3 times/wk) intake of													
- Sweetened milk	638	33			105	31.6			533	33.3			0.533
- Sugar sweetened beverages	735	38			134	40.4			601	37.5			0.339
- Dessert with coconut milk	174	9.0			31	9.3			143	8.9			0.835
Fruits and vegetables intake (portion/day)			2.63	1.95			2.62	2.04			2.63	1.93	0.910
**Sedentary activity time**													
- Total screen time (h/wk)			33.31	19.72			32.11	19.35			33.57	19.79	0.227
- TV^d^ viewing on weekdays (h/d)			2.39	1.56			2.33	1.65			2.41	1.55	0.402
- TV viewing on weekends (h/d)			3.78	2.63			3.77	2.65			3.80	2.62	0.847
- Use of computers, smart phones, tablets (h/wk)			14.59	13.52			14.24	13.40			14.66	13.54	0.626
- Educational screen time (h/wk)			2.40	2.68			2.17	2.52			2.45	2.71	0.092
**Physical activity time** (h/wk)			8.46	7.73			8.42	7.03			8.47	7.88	0.907
**Screen exposure at age ≤ 2 y**	902	46.6			166	50			736	45.9			0.260
**Laboratory results**													
Glucose (mg/dL)			89.43	12.00			93.47	20.81			88.60	11.97	< 0.001
Total cholesterol (mg/dL)			169.85	35.32			169.90	35.71			168.84	35.25	0.977
HDL-C^e^ (mg/dL)			51.07	12.49			40.58	8.25			53.24	12.11	< 0.001
LDL-C^f^ (mg/dL)			109.92	31.55			116.17	33.54			108.63	30.98	< 0.001
Triglyceride (mg/dL)			94.84	47.58			142.45	61.39			84.98	37.23	< 0.001

^a^ Classified as having metabolic syndrome by at least 1 out of 3 definitions

^b^ Body mass index z-score

^c^ Height-for-age z-score

^d^ Television

^e^ High-density lipoprotein cholesterol

^f^ Low-density lipoprotein cholesterol

The prevalence of metabolic syndrome by definition of IDF, Cook’s and de Ferranti’s were 4.1, 8.0, and 16.8%, respectively. Substantial agreement was found between the IDF and Cook’s definitions (Cohen kappa = 0.635). The agreement between Cook’s and de Ferranti’s definitions was moderate (Cohen kappa = 0.576). The IDF and de Ferranti’s definitions had the lowest agreement (Cohen kappa = 0.343). Among obese adolescents, the prevalence of metabolic syndrome was 23.0% by IDF, 37.2% by Cook’s, and 60.1% by de Ferranti’s definition.

The prevalence of high blood pressure was lowest in the IDF definition due to higher cut off level compared to the other two definitions. The systolic blood pressure was considered high according to IDF definition in 4.0% and diastolic blood pressure in 1.0% of participants. Only 2.7% of children aged 10–12 years had systolic or diastolic blood pressure at or above 130 and 85 mmHg. However, the proportion doubled among children aged 13–16 years (5.8%). For Cook’s and de Ferranti’s definitions, the fasting glucose level was the least common component. The HDL-C criterion was most prevalent in de Ferranti’s definition. Using de Ferranti’s definition, almost half of the participants were classified as having low HDL-C. The blood pressure and HDL-C criteria were significantly more common in male than female adolescents. The prevalence of metabolic syndrome and its components classified by each definition are shown in Table 2.

**Table 2:** Prevalence of metabolic syndrome and its components by different definitions

Definitions	Total *N* = 1934		Male *N* = 973		Female *N* = 961		***p***-value
	n	%	n	%	n	%	
**International Diabetes Federation’s definition**							
**Metabolic syndrome** (WC^a^ criterion plus ≥2/4 components)	**80**	**4.1**	**47**	**4.8**	**33**	**3.4**	0.123
WC ≥ P90 or adult cut-off if lower (mandatory criterion)	334	17.3	170	17.5	164	17.0	0.813
1. TG^b^ ≥ 150 mg/dL	212	11.0	109	11.2	103	10.7	0.733
2. HDL-C^c^ < 40 mg/dL	332	17.2	192	19.7	140	14.6	0.003
3. SBP^d^ ≥ 130 and/or DBP^e^ ≥ 85 mmHg	86	4.4	63	6.5	23	2.4	< 0.001
4. Fasting glucose ≥100 mg/dL	156	8.1	85	8.7	71	7.4	0.276
**Cook’s definition**							
**Metabolic syndrome (≥3/5 components)**	**155**	**8.0**	**88**	**9.0**	**67**	**7.0**	0.093
**1.** WC ≥ P90	325	16.8	169	17.4	156	16.2	0.504
**2.** TG ≥ 110 mg/dL	522	27.0	249	25.6	273	28.4	0.163
**3.** HDL-C ≤ 40 mg/dL	386	20.0	223	22.9	163	17.0	0.001
**4.** SBP and/or DBP ≥ P90	355	18.4	221	22.7	134	13.9	< 0.001
**5.** Fasting glucose ≥110 mg/dL	62	3.2	35	3.6	27	2.8	0.326
**de Ferranti’s definition**							
**Metabolic syndrome (≥3/5 components)**	**324**	**16.8**	**179**	**18.4**	**145**	**15.1**	0.051
1. WC > P75	630	32.6	306	31.4	324	33.7	0.288
2. TG ≥ 100 mg/dL	684	35.4	334	34.3	350	36.4	0.336
3. HDL-C < 50 mg/dL (< 45 mg/dL for boys aged 15–16)	895	46.3	474	48.7	421	43.8	0.030
4. SBP and/or DBP > P90	304	15.7	189	19.4	115	12.0	< 0.001
5. Fasting glucose ≥110 mg/dL	62	3.2	35	3.6	27	2.8	0.326

^a^ Waist circumference

^b^ Triglyceride

^c^ High-density lipoprotein cholesterol

^d^ Systolic blood pressure

^e^ Diastolic blood pressure

The combinations of metabolic syndrome components were classified into 11 different patterns by IDF definition, and 16 patterns for Cook’s and de Ferranti’s definitions. The most common pattern among all three definitions was the combination of the waist circumference, HDL-C, and triglyceride criteria, which was found in 40, 22.6 and 43.5% of participants with metabolic syndrome by IDF, Cook’s. and de Ferranti’s definition, respectively. There was no statistically significant difference between the occurrence of each pattern among male and female adolescents. (Supplementary Table S1).

From multivariable logistic regression analysis, age was negatively associated with metabolic syndrome by 1 out of 3 definitions (OR 0.88, 95% CI. 0.82–0.95) and de Ferranti’s definition (OR 0.89, 95% CI. 0.83–0.95). Male adolescents had significantly increased odds of metabolic syndrome by Cook’s (OR 1.46, 95% CI. 1.01–2.10) and de Ferranti’s definition (OR 1.44, 95% CI. 1.11–1.87). Participants who were exposed to screen media during the first 2 years of life was found to have an increased risk of having metabolic syndrome by at least one definition (OR 1.3, 95% CI. 1.01–1.68). Duration of physical activity was related to lower odds of metabolic syndrome by IDF definition (OR 0.96, 95% CI. 0.92–0.99). The association between these factors and metabolic syndrome are shown in Table 3. There were no significant association between frequency of intake of any listed food items, and amount of fruits and vegetables intake with metabolic syndrome.

**Table 3:** Risk factors and their association with metabolic syndrome

Factor	Odds ratio (95% CI)			
	MetS by any 1 of 3 definitions	MetS by IDF definition	MetS by Cook’s definition	MetS by de Ferranti’s definition
**Age**	**0.88**^a^ (0.82–0.95)	**0.95** (0.83–1.09)	**0.91** (0.83–1.01)	**0.89**^a^ (0.83–0.95)
**Male sex**	**1.44** (1.11–1.87)	**1.59** (0.95–2.65)	**1.46**^a^ (1.01–2.10)	**1.44**^a^ (1.11–1.87)
**Screen exposure at age ≤ 2 y**	**1.30**^a^ (1.01–1.68)	**1.18** (0.72–1.94)	**1.29** (0.90–1.85)	**1.27** (0.98–1.65)
**Total screen time** (h/wk)	**1.00** (0.99–1.00)	**1.00** (0.99–1.02)	**0.99** (0.98–1.00)	**1.00** (0.99–1.00)
**Physical activity time** (h/wk)	**0.98** (0.96–1.00)	**0.96**^a^ (0.92–0.99)	**0.98** (0.95–1.01)	**0.98** (0.97–1.00)
**Frequency of food intake** (> 3 vs ≤3 times/wk)				
- Sweetened milk	**0.84** (0.64–1.12)	**0.86** (0.49–1.48)	**0.88** (0.60–1.31)	**0.80** (0.60–1.06)
- Sugar sweetened beverages	**1.09** (0.83–1.44)	**1.16** (0.68–1.97)	**1.29** (0.88–1.88)	**1.09** (0.83–1.44)
- Dessert with coconut milk	**0.88** (0.56–1.39)	**1.12** (0.49–2.57)	**0.79** (0.41–1.52)	**0.91** (0.57–1.43)
**Amount of fruit and vegetable intake** (portion/day)	**1.05** (0.41–2.68)	**1.10** (0.98–1.24)	**1.02** (0.93–1.12)	**1.03** (0.96–1.10)

^a^ *p* < 0.05

## Discussion

This is the first study reporting Thailand’s national data on metabolic syndrome among adolescents. In previous studies, prevalence of metabolic syndrome in adolescents varies by definitions, age, sex, and ethnicities [17–21]. A multicenter cross-sectional study among adolescents aged 10–15 years in China and Spain found lower prevalence of metabolic syndrome by IDF definition compared to our study [17]. The prevalence of metabolic syndrome was only 0.5% in China and 2.5% in Spain, while in our study it was 4.1%. However, the waist circumference cut-off for adults was used, which can contribute to lower prevalence of metabolic syndrome compared to using the pediatric percentile cut-off. Another recently published national data from China [18], using pediatric waist circumference references, revealed the prevalence of metabolic syndrome by IDF definition of 2.5%. This was comparable to Korean [19] and Taiwanese [20] national studies, which reported the metabolic syndrome prevalence of 2.1 and 3.0% respectively. The national data from the United States in 2011–2016 [21] showed similar prevalence of metabolic syndrome by IDF definition (4.2%) as in Thai adolescents (4.1%). However, when using Cook’s and de Ferranti’s definitions, the prevalence of metabolic syndrome was lower in American (3.7 and 10.1%) than Thai adolescents (8.0 and 16.8%). A study in urban Vietnamese adolescents [22] also revealed comparable prevalence of metabolic syndrome by IDF definition (4.6%) to Thai adolescents and lower prevalence of metabolic syndrome by Cook’s and de Ferranti’s definitions (6.3 and 12.5%).

In our study, younger adolescents had higher risk of metabolic syndrome. This finding is consistent to a previous study in children and adolescents aged 7–17 years in Guangzhou, China [23], which found that the prevalence of metabolic syndrome was highest among adolescents aged 10–12 years. Another study in South Africa [24] also reported the inverse relationship between age and metabolic syndrome among adolescents aged 13–18 years. Insulin resistance was proposed as the central mechanism in the development of metabolic syndrome. Insulin sensitivity has been shown to decline transiently during puberty and can lead to impaired fasting plasma glucose [25, 26]. Changes in serum lipid profile also occur during puberty with the tendency of increasing serum triglyceride and decreasing HDL-C [27], contributing to the manifestation of metabolic syndrome during this period. In this study, the overall prevalence of metabolic syndrome was found to be highest at age 11 years in both male and female. This age is close to the previously reported average timing of puberty in Thai adolescents at around age 10 in female and 11–12 in male [28, 29]. The study by Cook et al. [10] also found that the prevalence of metabolic syndrome increased during early (Tanner stage 2–3) and decreased during late puberty (Tanner stage 4–5). However, studies had yielded inconsistent results on the association between age and metabolic syndrome in adolescents. The study by Messiah et al. [30] in the United States during 1999–2002 found that the prevalence of metabolic syndrome using Cook’s definition was only 1.2% in children aged 8–11 years and increased to 8.6% in those aged 12–14 years. Some other studies [18, 31] also reported the increasing risk of metabolic syndrome with age. The more prolonged course of obesity may contribute to higher occurrence of metabolic syndrome among older adolescents. Furthermore, the inconsistency could be due to the differences in race and the variation of the timing of puberty. Due to the potential effect of puberty-associated insulin resistance, the age range and pubertal status of the participants enrolled in each study can affect the prevalence of metabolic syndrome. Transient increase in the prevalence can occur among adolescents undergoing pubertal development.

The prevalence of metabolic syndrome in our study was higher in male than female adolescents. The same finding was also found in Chinese and American adolescents [18, 21]. However, previous report in Thai adults [32] revealed that metabolic syndrome was more prevalent among female than male, suggesting that age and pubertal stages might also play roles in the distribution of metabolic syndrome among both sexes.

The prevalence of metabolic syndrome among adolescents in Thailand was previously reported in a couple of studies from a single district and province. The prevalence of metabolic syndrome in the current study was higher than the report from Ubon Ratchathani province [14]. The prevalence of metabolic syndrome in Ubon Ratchathani was 3.1, 5.8, and 11.2% by IDF, Cook’s, and de Ferranti’s definitions, compared to 4.1, 8.0 and 16.8% in our study. The differences in metabolic syndrome prevalence could be due to regional variation and also the lower rate of obesity in the study from Ubon Ratchathani (5.1%) compared to our study (15.3%). Approximately 27% of the adolescents in that study were recruited from a local sports school and had significantly better metabolic profile than the rest of the participants recruited from conventional schools. In the same study, male adolescents was also found to be more predisposed to metabolic syndrome. Another study from Ongkhaluck district [13] found the prevalence of metabolic syndrome by de Ferranti’s definition of only 4%, which was substantially lower than that of our study (16.8%). The reason could be that serum HDL-C was not evaluated in the study, therefore the diagnosis was based on only four criteria.

The most common combination of metabolic syndrome components found in Thai adolescents consisted of waist circumference, HDL-C, and triglyceride. The same pattern was also identified as the most prevalence pattern among Thai adults [32].

In our study, we found that screen media exposure during the first 2 years of life was independently associated with increased risk of metabolic syndrome during adolescence. Screen media exposure during the first few years of life had been reported to be associated with increased BMI in later childhood [33, 34], but there is negligible evidence regarding the risk of developing metabolic syndrome. Our finding suggests that the recommendation to avoid screen time in children younger than two years of age should be reinforced. Early childhood is the critical period for the development of life style habits that can persist in later life and have long term effects. Therefore, health supervision during this period is crucial in the prevention of future health risks.

Many studies had reported the increased risk of metabolic syndrome with increasing screen time [35–37]. However, we found no differences in the total duration of screen time including television watching, smart phone, tablet, and computer use between those with and without metabolic syndrome. It might be due to the fact that there is small variation in the screen time in this population. Some previous studies also found no relationship between screen time and metabolic syndrome among children and adolescents [38, 39]. Another study in obese adolescents reported no association of self-reported screen time with cardiometabolic risk factors [40]. Previous studies had suggested that the relationship between children’s screen time and its metabolic impacts does not depend solely on the amount of time spent in front the screens but also the concomitant food consumption and exposure to food advertisements [41, 42].

We identified an inverse association between duration of physical activity and metabolic syndrome by IDF definition. Previous studies had suggested a protective role of physical activity against metabolic syndrome [38, 43–45]. Physical activity has also been shown to improve insulin sensitivity [46] and decrease inflammatory markers levels [47]. These findings emphasize the importance of promoting physical activity among children and adolescents for reduction of future cardiovascular risk.

We found no significant association between frequency of intake of sweetened milk, soft drinks or other sweetened beverages, and dessert with coconut milk with metabolic syndrome. However, intake of sugar sweetened beverages has been shown to be associated with metabolic syndrome in adolescents [48]. Added sugar was related to metabolic risk factors including obesity, abnormal lipid profile, hypertension, insulin resistance, and non-alcoholic fatty liver disease [49, 50]. High intake of saturated fatty acids has also been reported to associate with metabolic syndrome and its components [51–53].

In this study, there was no association between the amount of fruit and vegetable intake and metabolic syndrome. The reason could be that the reported fruits and vegetable intake were low in the majority of the participants. Over 70% of participants had only few portions of fruits and vegetable per day and less than 5% of them met the national daily recommendation. Some studies had found the inverse relationship between fruit and vegetable intake with metabolic syndrome and its components among adolescents [44, 54, 55]. However, a systematic review [56] suggested that there was inconsistency of the available evidences.

The limitations in this study include the cross-sectional design. Thus, the study cannot take into account the future metabolic consequences of the current lifestyle. There were 31.7% of NHES V subjects excluded from this study. The mean age of the excluded subjects was slightly lower than those included (12.82 (1.93) vs 13.40 (1.94) years). However, the sex distribution, obesity status, and area of residence were similar to those included in the analysis. Food intake in this study was measured as frequency of consumption which may not reflect amount of intake. The screen time and physical activity time was derived from self- and parent- reported data and there could be some under- or over-reporting. Regarding screen media use, the child and their parents or caretaker responded to the interview together as these are not sensitive questions. A series of questions were asked for each screen device including mobile phone, computer, tablet, and television. For each device, the questions on whether or not the child had used it, then the duration of screen time, and age at first exposure were asked. This process might help the respondents recall the data better than using a single question. The strengths of this study include the national representative sample and a relatively complete measurements of metabolic syndrome components.

## Conclusion

The prevalence of metabolic syndrome in the current study are higher than previously reported. Exposure to screen media during the first 2 years of life was associated with metabolic syndrome. Physical activity has a protective relationship with metabolic syndrome. Measures to prevent early screen exposure in young children and promote physical activity should be strengthened.

## Supplementary Information

**Additional file 1: Supplementary Table S1.** Patterns of combinations of metabolic syndrome components.

## Data Availability

This study is a secondary analysis of the data obtained from the 5th Thai National Health Examination Survey. The dataset and the questionnaire supporting the conclusions are available from the corresponding author on reasonable request.

## Abbreviations

BMI: Body mass index
HDL-C: High density lipoprotein cholesterol
IDF: International Diabetes Federation
MetS: Metabolic syndrome
NCEP-ATP III: Third report of National Cholesterol Education Program expert panel on detection, evaluation, and treatment of high blood cholesterol in adults
NHES V: National Health Examination Survey V
OR: Odds ratio
LDL-C: Low density lipoprotein cholesterol
