# Supplementary material for: Metabolic syndrome in Thai adolescents and associated factors: the Thai National Health Examination Survey V (NHES V)
Source: BMC Public Health. 2021 Apr 7;21:678. doi: 10.1186/s12889-021-10728-6 (PMC8028250; doi:10.1186/s12889-021-10728-6)
Supplement: Supplementary file 1 — Additional file 1: Supplementary Table S1. Patterns of combinations of metabolic syndrome components. [file 12889_2021_10728_MOESM1_ESM.docx]

**Supplementary table S1:** Patterns of combinations of metabolic syndrome components

| **Classification** | Components | | | | | Total | | Male | | Female | | p-value |
| --- | --- | --- | --- | --- | --- | --- | --- | --- | --- | --- | --- | --- |
|  | WC^a^ | TG^b^ | HDL^c^ | BP^d^ | BG^e^ | n | % | n | % | n | % |  |
| IDF  (N=80) | ✔ | ✔ | ✔ | ✔ | ✔ | 1 | 1.3 | 1 | 2.1 | 0 | 0 | 1.00 |
|  | ✔ | - | ✔ | ✔ | ✔ | 1 | 1.3 | 1 | 2.1 | 0 | 0 | 1.00 |
|  | ✔ | ✔ | - | ✔ | ✔ | 3 | 3.8 | 3 | 6.4 | 0 | 0 | 0.27 |
|  | ✔ | ✔ | ✔ | - | ✔ | 4 | 5.0 | 2 | 4.3 | 2 | 6.0 | 1.00 |
|  | ✔ | ✔ | ✔ | ✔ | - | 6 | 7.5 | 4 | 8.5 | 2 | 6.0 | 1.00 |
|  | ✔ | ✔ | ✔ | - | - | 32 | 40.0 | 18 | 38.3 | 14 | 42.4 | 0.82 |
|  | ✔ | ✔ | - | ✔ | - | 4 | 5.0 | 3 | 6.4 | 1 | 3.0 | 0.64 |
|  | ✔ | ✔ | - | - | ✔ | 4 | 5.0 | 2 | 4.3 | 2 | 6.0 | 1.00 |
|  | ✔ | - | ✔ | ✔ | - | 12 | 15.0 | 8 | 17.0 | 4 | 12.1 | 0.75 |
|  | ✔ | - | ✔ | - | ✔ | 10 | 12.5 | 3 | 6.4 | 7 | 21.2 | 0.08 |
|  | ✔ | - | - | ✔ | ✔ | 3 | 3.8 | 2 | 4.3 | 1 | 3.0 | 1.00 |
| Cook’s  (N = 155) | ✔ | ✔ | ✔ | ✔ | ✔ | 1 | 0.6 | 1 | 1.1 | 0 | 0 | 1.00 |
|  | ✔ | - | ✔ | ✔ | ✔ | 1 | 0.6 | 0 | 0 | 1 | 1.5 | 0.43 |
|  | ✔ | ✔ | - | ✔ | ✔ | 3 | 1.9 | 2 | 2.3 | 1 | 1.5 | 1.00 |
|  | ✔ | ✔ | ✔ | - | ✔ | 3 | 1.9 | 1 | 1.1 | 2 | 3.0 | 0.58 |
|  | ✔ | ✔ | ✔ | ✔ | - | 34 | 21.9 | 20 | 22.7 | 14 | 20.9 | 0.79 |
|  | ✔ | ✔ | ✔ | - | - | 35 | 22.6 | 21 | 23.9 | 14 | 20.9 | 0.66 |
|  | ✔ | ✔ | - | ✔ | - | 29 | 18.7 | 15 | 17 | 14 | 20.9 | 0.54 |
|  | ✔ | ✔ | - | - | ✔ | 1 | 0.6 | 1 | 1.1 | 0 | 0 | 1.00 |
|  | ✔ | - | ✔ | ✔ | - | 18 | 11.6 | 9 | 10.2 | 9 | 13.5 | 0.54 |
|  | ✔ | - | ✔ | - | ✔ | 3 | 1.9 | 0 | 0 | 3 | 4.5 | 0.08 |
|  | ✔ | - | - | ✔ | ✔ | 1 | 0.6 | 1 | 1.1 | 0 | 0 | 1.00 |
|  | - | ✔ | ✔ | ✔ | ✔ | 0 | 0 | 0 | 0 | 0 | 0 | - |
|  | - | ✔ | ✔ | ✔ | - | 22 | 14.2 | 14 | 15.9 | 8 | 11.9 | 0.48 |
|  | - | ✔ | ✔ | - | ✔ | 1 | 0.6 | 0 | 0 | 1 | 1.5 | 0.43 |
|  | - | ✔ | - | ✔ | ✔ | 3 | 1.9 | 3 | 3.4 | 0 | 0 | 0.26 |
|  | - | - | ✔ | ✔ | ✔ | 0 | 0 | 0 | 0 | 0 | 0 | - |
| De Ferranti’s  (N = 324) | ✔ | ✔ | ✔ | ✔ | ✔ | 3 | 0.9 | 2 | 1.1 | 1 | 0.7 | 1.00 |
|  | ✔ | - | ✔ | ✔ | ✔ | 0 | 0 | 0 | 0 | 0 | 0 | - |
|  | ✔ | ✔ | - | ✔ | ✔ | 1 | 0.3 | 1 | 0.6 | 0 | 0 | 1.00 |
|  | ✔ | ✔ | ✔ | - | ✔ | 5 | 1.5 | 3 | 1.7 | 2 | 1.4 | 1.00 |
|  | ✔ | ✔ | ✔ | ✔ | - | 74 | 22.8 | 45 | 25.1 | 29 | 20 | 0.27 |
|  | ✔ | ✔ | ✔ | - | - | 141 | 43.5 | 71 | 39.7 | 70 | 48.3 | 0.12 |
|  | ✔ | ✔ | - | ✔ | - | 15 | 4.6 | 7 | 3.9 | 8 | 5.5 | 0.49 |
|  | ✔ | ✔ | - | - | ✔ | 4 | 1.2 | 3 | 1.7 | 1 | 0.7 | 0.63 |
|  | ✔ | - | ✔ | ✔ | - | 43 | 13.3 | 27 | 15.1 | 16 | 11.0 | 0.29 |
|  | ✔ | - | ✔ | - | ✔ | 7 | 2.2 | 2 | 1.1 | 5 | 3.4 | 0.25 |
|  | ✔ | - | - | ✔ | ✔ | 0 | 0 | 0 | 0 | 0 | 0 | - |
|  | - | ✔ | ✔ | ✔ | ✔ | 1 | 0.3 | 1 | 0.6 | 0 | 0 | 1.00 |
|  | - | ✔ | ✔ | ✔ | - | 24 | 7.4 | 14 | 7.8 | 10 | 6.9 | 0.75 |
|  | - | ✔ | ✔ | - | ✔ | 5 | 1.5 | 2 | 1.1 | 3 | 2.1 | 0.66 |
|  | - | ✔ | - | ✔ | ✔ | 1 | 0.3 | 1 | 0.6 | 0 | 0 | 1.00 |
|  | - | - | ✔ | ✔ | ✔ | 0 | 0 | 0 | 0 | 0 | 0 | - |

^a^ Waist circumference

^b^ Triglyceride

^c^ High-density lipoprotein cholesterol

^d^ Blood pressure

^e^ Blood glucose
